# Validation of the ONKOTEV Risk Prediction Model for Venous Thromboembolism in Outpatients With Cancer

**DOI:** 10.1001/jamanetworkopen.2023.0010

**Published:** 2023-02-16

**Authors:** Chiara A. Cella, Maren Knoedler, Marcia Hall, Michele Arcopinto, Vincenzo Bagnardi, Lorenzo Gervaso, Stefania Pellicori, Francesca Spada, Maria G. Zampino, Paola S. Ravenda, Samuele Frassoni, Antonio Passaro, Monica Milano, Alice Laffi, Nicola Fazio, Florian Lordick

**Affiliations:** 1 Division of Gastrointestinal Medical Oncology and Neuroendocrine Tumors, European Institute of Oncology, IRCCS, Milan, Italy; 2 University Cancer Center Leipzig, University Hospital Leipzig, Leipzig, Germany; 3 Department of Medical Oncology, Mount Vernon Center for Cancer Treatment, Mount Vernon Hospital, Northwood, United Kingdom; 4 Department of Translational Medical Sciences, “Federico II” University Hospital and School of Medicine, Naples, Italy; 5 Department of Statistics and Quantitative Methods, University of Milan-Bicocca, Milan, Italy; 6 Molecular Medicine Department, University of Pavia, Pavia, Italy; 7 Oncology Department, Azienda Ospedaliera di Lodi, Lodi, Italy; 8 Division of Thoracic Oncology, European Institute of Oncology, IRCCS, Milan, Italy; 9 Division of Medical Senology, European Institute of Oncology IRCCS, Milan, Italy; 10 Medical Oncology and Hematology Unit, Humanitas Cancer Center, IRCCS Humanitas Research Hospital, Rozzano, Milan, Italy

## Abstract

**Importance:**

The assessment of the risk of venous thromboembolism (VTE) among outpatients with cancer represents an unsolved topic. Current international guidelines recommend primary prophylaxis for patients at intermediate to high risk of VTE, indicated by a Khorana score of 2 or more. A previous prospective study developed the ONKOTEV score, a 4-variable risk assessment model (RAM) consisting of a Khorana score of more than 2, metastatic disease, vascular or lymphatic compression, and previous VTE event.

**Objective:**

To validate the ONKOTEV score as a novel RAM to assess the risk of VTE among outpatients with cancer.

**Design, Setting, and Participants:**

ONKOTEV-2 is a noninterventional prognostic study conducted in 3 European centers located in Italy, Germany, and the United Kingdom among a prospective cohort of 425 ambulatory patients with a histologically confirmed diagnosis of a solid tumor who were receiving active treatments. The total study duration was 52 months, with an accrual period of 28 months (from May 1, 2015, to September 30, 2017) and an overall follow up-period of 24 months (data were censored September 30, 2019). Statistical analysis was performed in October 2019.

**Exposures:**

The ONKOTEV score was calculated for each patient at baseline by collecting clinical, laboratory, and imaging data from tests performed for routine practice. Each patient was then observed to detect any thromboembolic event throughout the study period.

**Main Outcomes and Measures:**

The primary outcome of the study was the incidence of VTE, including deep vein thrombosis and pulmonary embolism.

**Results:**

A total of 425 patients (242 women [56.9%]; median age, 61 years [range, 20-92 years]) were included in the validation cohort of the study. The cumulative incidences for the risk of developing VTE at 6 months were 2.6% (95% CI, 0.7%-6.9%), 9.1% (95% CI, 5.8%-13.2%), 32.3% (95% CI, 21.0%-44.1%), and 19.3% (95% CI, 2.5%-48.0%), respectively, among 425 patients with an ONKOTEV score of 0, 1, 2, and greater than 2 (*P* < .001). The time-dependent area under the curve at 3, 6, and 12 months was 70.1% (95% CI, 62.1%-78.7%), 72.9% (95% CI, 65.6%-79.1%), and 72.2% (95% CI, 65.2%-77.3%), respectively.

**Conclusions and Relevance:**

This study suggests that, because the ONKOTEV score has been validated in this independent study population as a novel predictive RAM for cancer-associated thrombosis, it can be adopted into practice and into clinical interventional trials as a decision-making tool for primary prophylaxis.

## Key Points

**Question:**

Is cancer-associated thrombosis risk among ambulatory patients with active cancer effectively assessed by the ONKOTEV score?

**Findings:**

In this prognostic study, the 4 ONKOTEV score levels were able to stratify the risk of venous thromboembolism (VTE) among outpatients with cancer.

**Meaning:**

This study suggests that the good stratification of the risk of VTE using the ONKOTEV score, together with the suitability and the affordability of variables used to calculate the score, could represent a breakthrough in cancer-associated thrombosis and the rationale for choosing the ONKOTEV score for risk assessment in the future.

## Teaser

This prognostic study assesses the ONKOTEV score as a novel risk assessment model to evaluate the risk of venous thromboembolism (VTE) among outpatients with cancer.

## Introduction

Venous thromboembolism (VTE) is a common cause of morbidity among patients with cancer, with a broad incidence range, and represents the most frequent cause of death in this population, after cancer itself.^1,2,3^ However, multiple risk factors (cancer, patient, and treatment related) can affect the individual risk of VTE.^4^ The assessment of cancer-associated thrombosis (CAT) risk and the recommendations about primary thromboprophylaxis for outpatients with cancer still represent an evolving topic. Several randomized clinical trials investigating the efficacy of primary thromboprophylaxis with low-molecular-weight heparins in the ambulatory cancer care setting have been reported, such as the PROTECHT, the PROSPECT-CONKO 004, the SAVE ONCO, the FRAGEM, and the TOPIC-1 and 2 trials.^5,6,7,8,9^ Despite the fact that these trials highlighted the benefit of primary prophylaxis in preventing thrombotic events among outpatients with cancer, VTE prophylaxis in an unselected population of patients with cancer has never been systematically adopted, because of unfavorable cost effectiveness and an unclear benefit-risk ratio. Different approaches to stratify the risk of VTE with risk assessment models (RAMs) have emerged, including the Khorana score, the Vienna Cancer and Thrombosis Score (CATS), the PROTECHT score, the CONKO score and—more recently—the COMPASS-CAT model.^10,11,12,13,14^ The Khorana score has been validated in multiple settings and is recommended by most international guidelines.^15,16,17,18^ The Vienna CATS, PROTECHT score, and CONKO score were developed to further improve the prediction of VTE by adding other parameters, such as soluble biomarkers (eg, D-dimer or P-selectin) or treatment-related variables (eg, type of chemotherapy). Overall, the aforementioned clinical prediction scores have a moderate to good ability to predict VTE.^10,11,12,13^ However, a direct comparison between the 4 scoring systems highlighted the need for a better refinement.^19^ The previously reported prospective ONKOTEV study developed a RAM for ambulatory patients with cancer consisting of 4 variables: a Khorana score greater than 2, metastatic disease, vascular or lymphatic compression, and previous VTE.^20^ On this basis, the primary goal of this study was to validate the ONKOTEV score in an independent prospective cohort.

## Methods

### Study Design and Participants

ONKOTEV-2 is a multicenter, prospective, noninterventional study designed to validate a clinical risk prediction model for VTE (ONKOTEV score), in a prospective cohort of ambulatory patients with cancer. The study was conducted in 3 centers: the European Institute of Oncology (IEO) in Milan (Italy), the University Cancer Center Leipzig (Germany), and the East and North Hertfordshire, National Health System Trust, in Stevenage (United Kingdom). The study was approved by the ethics committee of each center. Data were collected from medical records, transferred into a single clinical database, and analyzed at the coordinating center in Milan. Patients were enrolled at the participating centers between May 1, 2015, and September 30, 2017. The total study duration was 52 months, with an accrual period of 28 months and an overall follow-up period of 24 months (data were censored September 30, 2019). Written informed consent was obtained from study participants at baseline. This report adheres to the Transparent Reporting of a Multivariable Prediction Model for Individual Prognosis or Diagnosis (TRIPOD) reporting guideline.

The ONKOTEV score was calculated for each patient at baseline before any antitumor treatment, by collecting clinical, laboratory, and imaging data. Each patient was then clinically monitored for at least 8 months to detect any thromboembolic event. Patients were required to have a histologically confirmed diagnosis of solid tumor at any stage, to be 18 years of age or older, and to provide written informed consent, in accordance with the Declaration of Helsinki.^21^ In addition, eligible patients were those at the start of a new anticancer treatment, including chemotherapy, targeted therapy, immunotherapy, and endocrine therapy. Major surgery and locoregional treatments, namely radiotherapy—alone or in combination with chemotherapy—and minimally invasive techniques (radiofrequency ablation, microwave ablation, radioembolization, selective internal radiotherapy, or high-intensity focused ultrasound) were also allowed. Patients receiving anticoagulation therapy, both low-molecular-weight heparins or direct oral anticoagulants, were excluded. The use of aspirin, ticlopidine, or clopidogrel was allowed. Patients with inactive cancers, defined as a disease status that does not require any of the aforementioned active treatments, were further excluded. No routine screening for cancer-associated thrombosis was carried out due to the pure noninterventional design of the trial and to reflect clinical conditions; however, a contrast-enhanced thorax and abdomen computed tomography scan was performed at baseline and then periodically repeated during the restaging of disease during the anticancer treatment, as part of the routine care practice. In any cases of clinical suspicion of VTE, an objective imaging assessment was instantly arranged to confirm or rule out the event. Duplex sonography and/or computed tomography were usually applied for the diagnosis of deep vein thrombosis or pulmonary embolism, according to the algorithms provided by the diagnostic and therapeutic guidelines in use in each institution. Furthermore, VTE events were adjudicated by a team of expert radiologists. We did not use any action to blind the assessment of the outcome to be predicted or the assessment of the predictors of the outcome.

### Statistical Analysis

Statistical analysis was performed in October 2019. The number of patients enrolled in the present study have been determined based on VTE incidence observed in the original prospective ONKOTEV cohort.^20^ Assuming a VTE incidence of approximately 10% in a mean follow-up of 12 months, and using the criterion of a minimum number of events per predictor equal to 10,^22^ a sample size of at least 400 patients was deemed to be sufficient for the construction of a risk model that includes up to 4 predictors (ie, the number of variables included in the ONKOTEV score).

Patients with at least 1 missing value among the 4 variables involved in the ONKOTEV score calculation or the outcome of the study were excluded from the analyses. Continuous data were reported as median values and ranges or IQRs. Categorical data were reported as counts and percentages. The ONKOTEV score was calculated for each patient, assigning 1 point to each of the 4 factors considered in the score (Khorana score >2, previous VTE, metastatic disease, and macroscopic vascular or lymphatic compression) and then summing the points. The primary outcome of the study was the incidence of VTE. The validation of the ONKOTEV score was obtained by performing the cumulative incidence function (CIF) of VTE stratified by the ONKOTEV score in 4 levels (0, 1, 2, and >2). The CIF of VTE was estimated according to methods described by Kalbfleisch and Prentice,^23^ considering death as a competing event. The Gray test was used to assess differences between the ONKOTEV score levels. The discrimination ability of the model was assessed using the time-dependent receiver operator characteristic curve and the area under the receiver operator characteristic curve (AUROC). A multivariable Fine-Gray regression model with the 4 variables involved in the ONKOTEV score calculation was performed to assess the association of each single factor with VTE risk. All *P* values were from 2-sided tests and results were deemed statistically significant at *P* < .05. All analyses were performed with the statistical software SAS, version 9.4 (SAS Institute Inc).

## Results

### Patient Characteristics

A total of 643 outpatients with cancer from the 3 European centers were initially evaluated. Among them, 170 patients were not included because the inclusion or exclusion criteria were not met (52 patients had end-stage liver disease and/or kidney failure, 41 had an indolent tumor not requiring antitumoral treatment at screening, 32 were receiving anticoagulation therapy before screening, 29 were found to have a benign disease at histology report, 10 did not sign the informed consent, and 6 patients were younger than 18 years). A total of 473 patients from the 3 European centers were initially screened for the study. We assigned 1 point to each of the 4 variables contributing to the calculation of the ONKOTEV score (Khorana score >2, metastatic disease stage, presence of encasement or direct infiltration of vascular or lymphatic structures by gross tumor, and positive history for previous thromboembolic events). The variables involved in the ONKOTEV score calculation are separately shown in eTable 1 in Supplement 1. Forty-eight patients were also excluded because they had at least 1 missing value among the 4 variables involved in the ONKOTEV score calculation or the outcome of the study (eFigure in Supplement 1). Thus, 425 patients (242 women [56.9%]; median age, 61 years [range, 20-92 years]) were included in the validation cohort (272 in the Italian cohort, 140 in the German cohort, and 13 in the UK cohort). Patient demographic characteristics and disease characteristics are listed in Table 1. The most represented tumors were breast (77 [18.1%]), gastroesophageal adenocarcinoma (70 [16.5%]), colon (54 [12.7%]), lung (47 [11.1%]), rectum (46 [10.8%]), and pancreatic cancers (32 [7.5%]). The median time elapsed from initial diagnosis to start of antitumor treatment was 3 months (IQR, 2-13 months). Chemotherapy was the most frequent treatment in the study population (391 patients [92.0%] had chemotherapy as the only ongoing treatment). A total of 289 patients (68.0%) had metastatic disease. Most patients presented with an ONKOTEV score of 0 (116 [27.3%]) or 1 (234 [55.1%]), while 64 patients (15.1%) had an ONKOTEV score of 2, and 10 patients (2.4%) had an ONKOTEV score of 3. Only 1 patient (0.2%) had an ONKOTEV score of 4. Patient demographic characteristics and disease characteristics, separated by centers, are reported in eTable 2 in Supplement 1. Overall, 54 VTEs (12.7%) were diagnosed; VTEs occurred with a rate of 1.1 event-times per 100 person-months (54 events per 4865 person-months). Of the 54 events (both deep vein thrombosis and pulmonary embolism), 29 were incidentally diagnosed by imaging assessment (26 in the Italian cohort and 3 in the German cohort; a single VTE event occurred in the UK cohort, but we did not obtain information about this event). Ninety-one patients (21.4%) died: 72 (16.9%) with death as a first event and 19 (4.5%) with VTE as a first event (eTable 3 in Supplement 1). Two deaths occurred within 30 days from the VTE event (in the Italian cohort). The CIFs for the risk of developing VTE by ONKOTEV scoring are shown in the Figure. A total of 4 VTEs (3.4%) were diagnosed among the 116 patients with an ONKOTEV score of 0, 26 VTEs (11.1%) were diagnosed among the 234 patients with an ONKOTEV score of 1, 21 VTEs (32.8%) were diagnosed among the 64 patients with an ONKOTEV score of 2, and 3 VTEs (27.3%) were diagnosed among the 11 patients with an ONKOTEV score of greater than 2.

**Table 1.** Patient Demographic Characteristics and Disease Characteristics

Characteristic	No. (%) (N = 425)
Age at start of the therapy, median (range), y	61 (20-92)
BMI, median (range)	24.4 (15.1-44.8)
Ongoing treatment	
Chemotherapy	391 (92.0)
Radiotherapy	3 (0.7)
Surgery	1 (0.2)
Concomitant chemotherapy and radiotherapy	13 (3.1)
Endocrine therapy	2 (0.5)
Target therapy	8 (1.9)
Chemotherapy and endocrine therapy	3 (0.7)
Sequential chemotherapy and radiotherapy	1 (0.2)
Chemotherapy and surgery	2 (0.5)
Locoregional treatment	1 (0.2)
Tumor site	
Colon	54 (12.7)
Rectum	46 (10.8)
Breast	77 (18.1)
Gastric or early gastric cancer	70 (16.5)
Lung	47 (11.1)
Pancreas	32 (7.5)
Biliary tract	13 (3.1)
Bladder or urinary tracts	3 (0.7)
Prostate	1 (0.2)
Mesothelioma	2 (0.5)
Head and neck	7 (1.6)
Gynecologic or urological	26 (6.1)
Anus	4 (0.9)
Sarcoma	2 (0.5)
Esophagus	7 (1.6)
Skin	2 (0.5)
Neuroendocrine tumor (thoracic and GEP)	24 (5.6)
Appendix	1 (0.2)
Unknown	7 (1.6)
Variables involved in Khorana score calculation and Khorana score	
Tumor risk	
Low	247 (58.1)
High	76 (17.9)
Very high	102 (24.0)
Hemoglobin level <10 g/dL or use of red blood cell growth factors	
No	401 (94.4)
Yes	24 (5.6)
Prechemotherapy leukocyte count >11 000/µL	
No	384 (90.4)
Yes	41 (9.6)
Prechemotherapy platelet count ≥350 × 10^3^/µL	
No	343 (80.7)
Yes	82 (19.3)
BMI ≥35	
No	408 (96.0)
Yes	17 (4.0)
Khorana score	
0	181 (42.6)
1	92 (21.6)
2	114 (26.8)
3	29 (6.8)
4	8 (1.9)
5	1 (0.2)
Variables involved in ONKOTEV score calculation and ONKOTEV score	
Khorana score	
≤2	387 (91.1)
>2	38 (8.9)
Previous venous thromboembolism	
No	394 (92.7)
Yes	31 (7.3)
Metastatic disease	
No	136 (32.0)
Yes	289 (68.0)
Macroscopic vascular or lymphatic compression	
No	387 (91.1)
Yes	38 (8.9)
ONKOTEV score	
0	116 (27.3)
1	234 (55.1)
2	64 (15.1)
3	10 (2.4)
4	1 (0.2)

Abbreviations: BMI, body mass index (calculated as weight in kilograms divided by height in meters squared); GEP, gastroenteropancreatic.

SI conversion factors: To convert hemoglobin to grams per liter, multiply by 10; leukocytes to ×10^9^/L, multiply by 0.001; and platelets to ×10^9^/L, multiply by 1.0.

**Figure.**
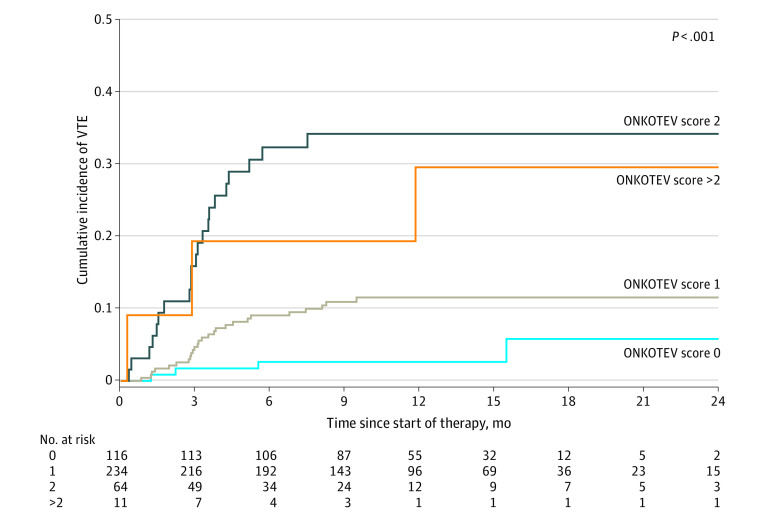
Cumulative Incidence Function for the Risk of Developing a Venous Thromboembolism (VTE) by the ONKOTEV Score in 4 Levels

The cumulative incidences for the risk of developing VTE at 6 months were 2.6% (95% CI, 0.7%-6.9%), 9.1% (95% CI, 5.8%-13.2%), 32.3% (95% CI, 21.0%-44.1%), and 19.3% (95% CI, 2.5%-48.0%), respectively, among patients with an ONKOTEV score of 0, 1, 2, and greater than 2 (*P* < .001). The time-dependent AUROC at 3, 6, and 12 months was 70.1% (95% CI, 62.1%-78.7%), 72.9% (95% CI, 65.6%-79.1%), and 72.2% (95% CI, 65.2%-77.3%), respectively. Multivariable Fine-Gray regression analysis with variables considered in the ONKOTEV score calculation is reported in Table 2. Metastatic disease (hazard ratio [HR], 4.22 (95% CI, 1.66-10.74; *P* = .003) and macroscopic vascular or lymphatic compression (HR, 3.25 [95% CI, 1.64-6.43]; *P* < .001) were found to be highly associated with VTE. Similarly, patients with a Khorana score greater than 2 seemed to have a higher risk of VTE, although the difference was not statistically significant (HR, 1.81 [95% CI, 0.82-3.97]; *P* = .14). Previous VTE did not appear to be independently associated with the risk of VTE (HR, 0.84 [95% CI, 0.28-2.52]; *P* = .76) in this validation cohort. A comparison of development and validation data is shown in eTable 4 in Supplement 1.

**Table 2.** Fine-Gray Multivariable Regression Model With Variables Involved in ONKOTEV Score Calculation^a^

Variable	Multivariable analysis	
	HR (95% CI)	*P* value
Khorana score		
≤2	1 [Reference]	.14
>2	1.81 (0.82-3.97)	
Metastatic disease		
No	[Reference]	.003
Yes	4.22 (1.66-10.74)	
Macroscopic vascular or lymphatic compression		
No	[Reference]	<.001
Yes	3.25 (1.64-6.43)	
Previous VTE		
No	[Reference]	.76
Yes	0.84 (0.28-2.52)	

Abbreviations: HR, hazard ratio; VTE, venous thromboembolism.

^a^ Outcome: VTE (54 events, 72 competing events).

## Discussion

Our study has validated the ONKOTEV score in an independent study population as a novel predictive RAM for cancer-associated thrombosis. The previously reported ONKOTEV study prospectively developed a 4-variable RAM incorporating the most significant category of the Khorana model, the Khorana score greater than 2.^20^ We added 3 new items to the Khorana score: metastatic disease stage, the presence of encasement or direct infiltration of vascular or lymphatic structures by gross tumor, and a positive history for previous thromboembolic events not requiring anticoagulation therapy at the start of the study. These additional 3 items were chosen based on published findings^3^ or clinical hypothesis.^20^ The Khorana score represents the most widely used RAM for the prevention of VTE in outpatients with cancer and is the only tool currently endorsed by international guidelines.^15,16,17,18,24,25^ Even though the CIF at 12 months of developing a VTE by the criterion of a Khorana score of 2 or more was not reported in the pivotal trial, the Khorana score has been subsequently validated in multiple settings. With a view of improving the discriminatory capabilities of the Khorana score, novel risk models were proposed in the past few years (such as the Vienna CATS, PROTECHT score, and CONKO score) and were characterized by the addition of soluble biomarkers (eg, D-dimer or P-selectin) or new items, such as chemotherapy type, or by replacing body mass index with performance status. More recently, the COMPASS-CAT model was validated by Pabinger et al^26^ in 2 independent prospective cohorts by including 1 clinical item (tumor site category) and 1 biomarker (D-dimer). However, although the results of these scoring models in each primary study are promising, they lack subsequent external validation or consistent results in independent cohorts, which represents a limitation to their routine use in clinical practice. The only direct comparative analysis of the 4 scoring systems (the Khorana, Vienna CATS, PROTECHT score, and CONKO score), carried out by van Es et al,^19^ showed that all scores had a poor discriminatory performance at the thresholds of 3 points, discouraging the use of any model for stratification of patients. The Vienna CATS and the PROTECHT score were able to discriminate patients at high and low risk when used dichotomously, although the 6-month incidence scores of VTE were not very high (9.1% and 9.6% for each score).^19^ Potential explanations for the poor stratification performance of the RAMs may be attributable to the presence of time-dependent variables, such as blood cells, that can be affected by ongoing medical treatments. Another prospective analysis of different RAMs (Khorana score, Vienna CATS, PROTECHT score, ONKOTEV score, and CATS score^26^) has been reported by Schorling et al^27^ in a subpopulation of ONKOTEV study patients (n = 100) recruited at the University Cancer Center Leipzig between August 2016 and March 2017. Finally, in a recent retrospective study, Di Nisio and colleagues^28^ evaluated the discriminatory capability of the Khorana, PROTECHT, CONKO, and ONKOTEV scores both at baseline and after 3 to 6 months from the start of treatment, to explore whether subsequent reassessment could affect the stratification performance over time. Consistently, the study confirmed the poor accuracy of all scores at the conventional 3-point threshold and highlighted the improvement in discriminatory performances, which improved at the 2-point threshold. Moreover, they showed that that RAM accuracy tends to decrease over time, suggesting the potential usefulness of periodic reassessment. Overall, all the available RAMs have some weakness in discriminatory power. So far, the Khorana score still remains the most widely used risk score in clinical practice as well as in clinical trials. For instance, the AVERT and CASSINI trials, which were designed to evaluate the efficacy and safety of direct oral anticoagulants as primary prophylaxis of cancer-associated thrombosis in outpatients with cancer at high risk, used a Khorana score cutoff of 2 or higher for patient selection.^29,30^ In the original validation study, the threshold for discriminating an intermediate- to high-risk group was 3 or more.^10^ The lower cutoff score in the AVERT and CASSINI trials was chosen independently by the investigators and was based on data reported by Ay et al^11^ in a population-based study, in which the 6-month CIF of VTE assessed by a Khorana score of 2 was nearly 10%. Accordingly, the ONKOTEV score of 2 or higher showed a higher CIF at several time points (6, 8, and 12 months) compared with the CIF reported in other RAMs. Furthermore, the ONKOTEV score is an easy-to-use and cost-effective model based on routinely collected clinical information, preventing the need to perform tests for highly selective biochemical parameters.

### Limitations

Our study presents certain limitations. First, the derivation and the validation populations are different and not properly balanced in terms of patient, tumor, and treatment characteristics. Second, the previous personal history of VTE events is not independently associated with the risk of VTE, contrary to the derivation cohort. This aspect could be associated with the low frequency rate of the variable in the study population and warrants subsequent analysis to define its real association with risk definition. Third, results from comparative analyses (both retrospective and prospective) are contrasting, highlighting the importance of further validating the ONKOTEV score in multiple prospective settings and populations.

## Conclusions

The ONKOTEV score has been validated as a novel RAM for assessing the risk of cancer-associated thrombosis among outpatients with cancer. The discriminatory performances, together with the suitability and the affordability of variables used, could represent a breakthrough in cancer-associated thrombosis and a rationale for choosing the ONKOTEV score for risk assessment in the future. Despite the need for further validation in different and heterogeneous settings, we envision a widespread application of the ONKOTEV score in clinical practice and in clinical trials for selecting patients at high risk of VTE who may benefit from primary prophylaxis.

## Notes

Supplement 1. **eFigure.** Flow Chart and Causes of Patients’ Exclusion **eTable 1.** Variables Involved in ONKOTEV Score Calculation (N=425) **eTable 2.** Patient Demographics and Disease Characteristics, Separated by Centers (N=425) **eTable 3.** Outcomes of the Study, Overall and by Centers (N=425) **eTable 4.** Comparison of Development and Validation Data

Supplement 2.
Data Sharing Statement
