# Supplementary material for: Validation of the ONKOTEV Risk Prediction Model for Venous Thromboembolism in Outpatients With Cancer
Source: JAMA Netw Open. 2023 Feb 16;6(2):e230010. doi: 10.1001/jamanetworkopen.2023.0010 (PMC9936336; doi:10.1001/jamanetworkopen.2023.0010)
Supplement: Supplement 1. — eFigure. Flow Chart and Causes of Patients’ Exclusion eTable 1. Variables Involved in ONKOTEV Score Calculation (N=425) eTable 2. Patient Demographics and Disease Characteristics, Separated by Centers (N=425) eTable 3. Outcomes of the Study, Overall and by Centers (N=425) eTable 4. Comparison of Development and Validation Data [file jamanetwopen-e230010-s001.pdf]

Supplementary Online Content

Cella CA, Knoedler M, Hall M, et al. Validation of the ONKOTEV risk prediction model for venous thromboembolism in outpatients with cancer. *JAMA Netw Open.* 2023;6(2):e230010. doi:10.1001/jamanetworkopen.2023.0010

eFigure. Flow Chart and Causes of Patients' Exclusion
eTable 1. Variables Involved in ONKOTEV Score Calculation (N=425)
eTable 2. Patient Demographics and Disease Characteristics, Separated by Centers (N=425)
eTable 3. Outcomes of the Study, Overall and by Centers (N=425)
eTable 4. Comparison of Development and Validation Data

This supplementary material has been provided by the authors to give readers additional information about their work.

© 2023 Cella CA et al. *JAMA Network Open.*

eFigure. Flow Chart and Causes of Patients' Exclusion

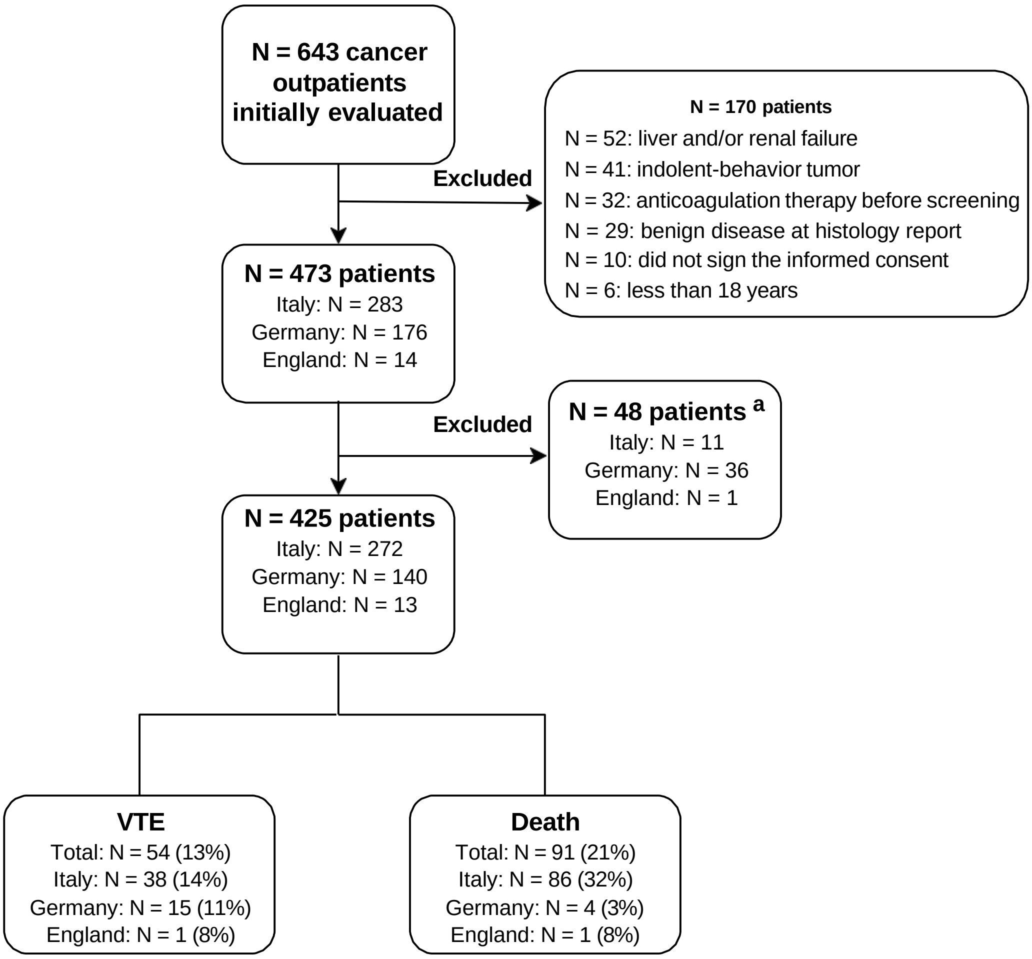

a. Causes of patients' exclusion

From	Ongoing treatment missing/None/SF	Date of start missing	ONKOTEV score missing	Outcome missing	Date of outcome missing	Overall, No. (%)
Italy			X			1 (2%)
Italy			X		X	1 (2%)
Italy		X	X			1 (2%)
Italy		X	X	X		1 (2%)
Italy	X	X	X			1 (2%)
Italy	X	X	X	X		6 (13%)
Germany					X	18 (38%)
Germany				X		17 (35%)
Germany	X	X	X	X		1 (2%)
England			X			1 (2%)

List of abbreviations: VTE: Venous thromboembolism; SF: Screening failure.

© 2023 Cella CA et al. *JAMA Network Open.*

eTable 1. Variables Involved in ONKOTEV Score Calculation (N=425)

Khorana Score	Previous VTE	Metastatic disease	Macroscopic vascular or lymphatic compression	ONKOTEV Score	Overall (n=425), No. (%)
≤2	No	No	No	0	116 (27.3)
≤2	No	No	Yes	1	6 (1.4)
≤2	No	Yes	No	1	214 (50.4)
≤2	Yes	No	No	1	8 (1.9)
>2	No	No	No	1	6 (1.4)
≤2	No	Yes	Yes	2	22 (5.2)
≤2	Yes	Yes	No	2	18 (4.2)
>2	No	Yes	No	2	24 (5.6)
≤2	Yes	Yes	Yes	3	3 (0.7)
>2	No	Yes	Yes	3	6 (1.4)
>2	Yes	Yes	No	3	1 (0.2)
>2	Yes	Yes	Yes	4	1 (0.2)

List of abbreviations: VTE: Venous thromboembolism.

© 2023 Cella CA et al. *JAMA Network Open.*

eTable 2. Patient Demographics and Disease Characteristics, Separated by Centers (N=425)

Variable	Level	Italy (n=272)	Germany (n=140)	England (n=13)
		No. (%)	No. (%)	No. (%)
**Age at start of the therapy, median (min-max), y**		62 (20-86)	60 (27-82)	66 (44-92)
**BMI, median (min-max), kg/m^2**		24.5 (15.1-43.5)	24.1 (16.0-44.8)	26.6 (19.9-37.5)
**Ongoing treatment**	CH	243 (89.3)	140 (100)	8 (61.5)
	RT	3 (1.1)	0 (0.0)	0 (0.0)
	Surgery	1 (0.4)	0 (0.0)	0 (0.0)
	Concomitant CH-RT	11 (4.0)	0 (0.0)	2 (15.4)
	Endocrine therapy	2 (0.7)	0 (0.0)	0 (0.0)
	Target therapy	8 (2.9)	0 (0.0)	0 (0.0)
	CH + Endocrine therapy	0 (0.0)	0 (0.0)	3 (23.1)
	Sequential CH-RT	1 (0.4)	0 (0.0)	0 (0.0)
	CH + Surgery	2 (0.7)	0 (0.0)	0 (0.0)
	Locoregional treatment	1 (0.4)	0 (0.0)	0 (0.0)
**Tumor site**	Colon	42 (15.4)	11 (7.9)	1 (7.7)
	Rectum	39 (14.3)	6 (4.3)	1 (7.7)
	Breast	27 (9.9)	46 (32.9)	4 (30.8)
	Gastric/EGC	53 (19.5)	17 (12.1)	0 (0.0)
	Lung	39 (14.3)	7 (5.0)	1 (7.7)
	Pancreas	25 (9.2)	7 (5.0)	0 (0.0)
	Biliary tract	5 (1.8)	8 (5.7)	0 (0.0)
	Bladder/urinary tracts	1 (0.4)	2 (1.4)	0 (0.0)
	Prostate	0 (0.0)	1 (0.7)	0 (0.0)
	Mesothelioma	1 (0.4)	1 (0.7)	0 (0.0)
	Head and neck	2 (0.7)	5 (3.6)	0 (0.0)
	Gynecologic/urological	1 (0.4)	20 (14.3)	5 (38.5)
	Anus	2 (0.7)	1 (0.7)	1 (7.7)
	Sarcoma	0 (0.0)	2 (1.4)	0 (0.0)
	Esophagus	4 (1.5)	3 (2.1)	0 (0.0)
	Skin	2 (0.7)	0 (0.0)	0 (0.0)
	NET (pulmonary and GEP)	23 (8.5)	1 (0.7)	0 (0.0)
	Appendix	0 (0.0)	1 (0.7)	0 (0.0)
	Unknown	6 (2.2)	1 (0.7)	0 (0.0)
Variables involved in Khorana score calculation and Khorana score				
**Tumor risk**	Low risk	153 (56.2)	87 (62.1)	7 (53.8)

© 2023 Cella CA et al. *JAMA Network Open.*

Variable	Level	Italy (n=272)	Germany (n=140)	England (n=13)
		No. (%)	No. (%)	No. (%)
	High risk	41 (15.1)	29 (20.7)	6 (46.2)
	Very high risk	78 (28.7)	24 (17.1)	0 (0.0)
**Hemoglobin level <100 g/L or use of red cell growth factors**	No	262 (96.3)	126 (90.0)	13 (100)
	Yes	10 (3.7)	14 (10.0)	0 (0.0)
**Prechemotherapy leukocyte count >11×10^9/L**	No	249 (91.5)	125 (89.3)	10 (76.9)
	Yes	23 (8.5)	15 (10.7)	3 (23.1)
**Prechemotherapy platelet count 350×10^9/L or greater**	No	241 (88.6)	93 (66.4)	9 (69.2)
	Yes	31 (11.4)	47 (33.6)	4 (30.8)
**BMI≥35**	No	266 (97.8)	130 (92.9)	12 (92.3)
	Yes	6 (2.2)	10 (7.1)	1 (7.7)
**Khorana score**	0	130 (47.8)	47 (33.6)	4 (30.8)
	1	48 (17.6)	40 (28.6)	4 (30.8)
	2	69 (25.4)	40 (28.6)	5 (38.5)
	3	20 (7.4)	9 (6.4)	0 (0.0)
	4	4 (1.5)	4 (2.9)	0 (0.0)
	5	1 (0.4)	0 (0.0)	0 (0.0)
Variables involved in ONKOTEV score calculation and ONKOTEV score				
**Khorana score**	≤2	247 (90.8)	127 (90.7)	13 (100)
	>2	25 (9.2)	13 (9.3)	0 (0.0)
**Previous VTE**	No	247 (90.8)	134 (95.7)	13 (100)
	Yes	25 (9.2)	6 (4.3)	0 (0.0)
**Metastatic disease**	No	64 (23.5)	65 (46.4)	7 (53.8)
	Yes	208 (76.5)	75 (53.6)	6 (46.2)
**Macroscopic vascular or lymphatic compression**	No	244 (89.7)	132 (94.3)	11 (84.6)
	Yes	28 (10.3)	8 (5.7)	2 (15.4)
**ONKOTEV score**	0	53 (19.5)	56 (40.0)	7 (53.8)
	1	164 (60.3)	66 (47.1)	4 (30.8)
	2	44 (16.2)	18 (12.9)	2 (15.4)
	3	10 (3.7)	0 (0.0)	0 (0.0)
	4	1 (0.4)	0 (0.0)	0 (0.0)

List of abbreviations: y: years, CH: chemotherapy, RT: radiotherapy, EGC: early gastric cancer, NET: neuroendocrine tumor, GEP: gastro-entero-pancreatic, VTE: venous thromboembolism.

© 2023 Cella CA et al. *JAMA Network Open.*

eTable 3. Outcomes of the Study, Overall and by Centers (N=425)

**Variable**	**Level**	**Overall (n=425)**	**Italy (n=272)**	**Germany (n=140)**	**England (n=13)**
		**No. (%)**	**No. (%)**	**No. (%)**	**No. (%)**
**VTE**	No	371 (87.3)	234 (86.0)	125 (89.3)	12 (92.3)
	Yes	54 (12.7)	38 (14.0)	15 (10.7)	1 (7.7)
**Death**	Alive	334 (78.6)	186 (68.4)	136 (97.1)	12 (92.3)
	Death	91 (21.4)	86 (31.6)	4 (2.9)	1 (7.7)
**VTE / Death**	No events	299 (70.4)	164 (60.3)	124 (88.6)	11 (84.6)
	VTE as first event	54 (12.7)	38 (14.0)	15 (10.7)	1 (7.7)
	Death as first event	72 (16.9)	70 (25.7)	1 (0.7)	1 (7.7)

List of abbreviations: VTE: Venous thromboembolism.

© 2023 Cella CA et al. *JAMA Network Open.*

eTable 4. Comparison of Development and Validation Data

Variable	Level	Development data (n=843)	Validation data (n=425)	P-value
		No. (%)	No. (%)	
**Tumor risk**	Low risk	595 (70.6)	247 (58.1)	<0.001
	High risk	162 (19.2)	76 (17.9)	
	Very high risk	86 (10.2)	102 (24.0)	
**Hemoglobin level <100 g/L or use of red cell growth factors**		112 (13.5)	24 (5.6)	<0.001
**Prechemotherapy leukocyte count >11×10^9/L**		56 (6.7)	41 (9.6)	0.058
**Prechemotherapy platelet count 350×10^9/L or greater**		121 (14.4)	82 (19.3)	0.024
**BMI≥35 kg/m^2**		53 (6.4)	17 (4.0)	0.092
**Khorana score**	0 (Low risk)	405 (49.8)	181 (42.6)	0.043
	1-2 (Intermediate risk)	352 (43.3)	206 (48.5)	
	>2 (High risk)	56 (6.9)	38 (8.9)	
**Previous VTE**		83 (9.8)	31 (7.3)	0.13
**Metastatic disease**		465 (55.2)	289 (68.0)	<0.001
**Macroscopic vascular or lymphatic compression**		69 (8.2)	38 (8.9)	0.65
**VTE** [a]		73 (8.6)	54 (12.7)	0.024

List of abbreviations: VTE: Venous thromboembolism.

a. Development data: The CIF (95% CI) at 12 months in patients with ONKOTEV score 0, 1, 2, and >2 is, respectively, 3.7 (1.1-6.3), 9.7 (6.5-12.9), 19.4 (10.1-28.7), and 33.9 (20.3-47.4).
Validation data: The CIF (95% CI) at 12 months in patients with ONKOTEV score 0, 1, 2, and >2 is, respectively, 2.6 (0.7-6.9), 11.5 (7.8-16.1), 34.2 (22.5-46.2), and 29.5 (4.7-61.6).

© 2023 Cella CA et al. *JAMA Network Open.*
