# Supplementary material for: Validation of the ONKOTEV Risk Prediction Model for Venous Thromboembolism in Outpatients With Cancer
Source: JAMA Netw Open. 2023 Feb 16;6(2):e230010. doi: 10.1001/jamanetworkopen.2023.0010 (PMC9936336; doi:10.1001/jamanetworkopen.2023.0010)
Supplement: Supplement 2. — Data Sharing Statement [file jamanetwopen-e230010-s002.pdf]

Data Sharing Statement

Cella. Validation of the ONKOTEV Risk Prediction Model for Venous Thromboembolism in Outpatients With Cancer. *JAMA Netw Open*. Published February 16, 2023. doi:10.1001/jamanetworkopen.2023.0010

Data
Data available: No

Additional Information
Explanation for why data not available: Data presented in this study are available on request from the corresponding authors.
